# Repeat Breeding and Its' Associated Risk Factors in Crossbred Dairy Cattle in Northern Central Highlands of Ethiopia

**DOI:** 10.1155/2023/1176924

**Published:** 2023-01-21

**Authors:** Tewodros Eshete, Tilaye Demisse, Tefera Yilma, Berhan Tamir

**Affiliations:** ^1^Department of Animal Science, College of Agriculture and Natural Resource Science, Debre Berhan University, Debre Berhan, Amhara, Ethiopia; ^2^Department of Veterinary Obstetrics and Gynecology, College of Veterinary Medicine and Agriculture, Addis Ababa University, P.O. Box 34, Hora Road, Bishoftu, Oromia, Ethiopia; ^3^Department of Animal Production Studies, College of Veterinary Medicine and Agriculture, Addis Ababa University, P.O. Box 34, Hora Road, Bishoftu, Oromia, Ethiopia

## Abstract

The purpose of the current study was to ascertain the prevalence and incidence of repeat breeding and related risk variables in crossbred dairy cattle in the northern Central Highlands of Ethiopia. The prevalence and incidence of repeat breeding in crossbred dairy cattle were 38.4% and 36.6%, respectively, out of the total cows and heifers taken into account for this study and handled with various production strategies. Age, parity, body condition, breeding practices, milk yield, management condition, and insemination time were all substantially (*P* < 0.05) associated with the prevalence and incidence of repeat breeders. Repeat breeder is more common in elderly, underweight, multiparous, high-milk producing, and AI serviced cows, as well as cows kept in poor management condition. It was deduced that the production system had a significant impact (*P* < 0.05) on the prevalence of repeat breeders in the research area. In addition, herd size was significantly associated (*P* < 0.05) with the prevalence of repeat breeder in this study. Insemination time and heat detection practices were also substantially (*P* < 0.05) interrelated with the prevalence and incidence of repeat breeders, indicating that incorrect heat detection and/or insemination timing had an impact on these variables. Therefore, it is suggested to increase the mindfulness of farm owners, managers, and attendants about appropriate feed and feeding management, accurate heat detection, and insemination time. To reduce the incidence of repeat breeders and the associated reproductive issues, as well as the resulting financial losses on dairy farms, health, housing, and reproductive management should also be improved.

## 1. Introduction

With 65.4 million heads, Ethiopia has a sizable herd of cattle [1]. This large cattle population plays multiple socio-economic roles and has religious and cultural values. Despite the massive number of cattle and their significance for society and the economy, dairy production is nevertheless a smallholder-based business that relies on smallholder survival [2]. This is due to a number of factors, including sickness, various reproductive issues, terrible nutrients, the low genetic potential of native breeds, and the traditional way of husbandry practices. A number of the fundamental reproductive issues which have an immediate effect on the reproductive performance of dairy cows are repeat breeder (RB), abortion, anoestrous, dystocia, retained fetal membrane (RFM), metritis, and prolapse (vaginal and uterine) [3–5]. By reducing milk output, increasing open days and calving intervals, failing uterine involution, and culling valuable cows early, reproductive issues have an impact on the fertility and productivity of dairy cows [4, 6].

According to definitions, a repeat breeder is a cow or heifer that is unable to become pregnant after three or more straight services with fertile semen or a bull, has a regular oestrous cycle, and is healthy and devoid of any defects that can be seen [7]. Regardless of the management practices, repeat breeding remains a major cause of infertility of dairy cows, economic losses because of wastage of valuable time, monetary loss, reduce lifetime milk yield of cow, replacement costs, and loss of genomic improvement [4]. The causes of RB are multifactorial [7, 8], which include inaccuracies in oestrous detection and insemination time, as well as the semen quality and insemination technique [9]. Furthermore, uterine infection [7], hormonal imbalance [10, 11], anovulatory heat, delayed ovulation, blocked fallopian tubes, and embryonic death are possibly the causes of RB [7]. In Ethiopia, the prevalence of RB ranges from 1.3% to 28% [5, 12–16].

In Ethiopia, studies on reproductive problems in dairy cattle are few and mostly concentrate on urban and peri-urban areas, despite the fact that these conditions are the subject of extensive research worldwide [5]. Furthermore, information from these studies, which were focused on identifying the major reproductive disorders, has not yet been well documented to indicate the extent of occurrences of repeat breeder crossbred dairy cows in Ethiopia. Due to expansion, population growth, increased demand for milk, and the employment of unemployed residents of the surrounding villages by dairy farms, production on small-scale and commercial dairy farms has expanded quickly. However, the performance of dairy cow and profitability of the dairy sector in the study area are extremely low. Moreover, to our knowledge, there is no detailed and well-documented research outcome on the occurrence and etiological factors of RB in the northern central highland of Ethiopia. Based on the above justification and background, the current study was conducted to determine the prevalence and incidence of RB, as well as to identify major etiological factors of RB in the study area.

## 2. Material and Methodology

### 2.1. Study Site

In the northern central highlands of Ethiopia, the study was carried out in milk shed locations (Debre Berhan, Chacha, Basonawerena, and Sheno). Districts in the north shoa zone of the Amhara national regional state include Debre Berhan, Basonaworena, and Chacha. The capital town of the zone is Debre Berhan, located 130 km away northeast of Addis Ababa. These districts are located between 9°34′ and 9°542′ north latitudes and 39°29′ and 39°44′ east longitudes with an elevation range from 2056–3744 m.a.s.l [17]. The mean monthly temperature of the study area ranges from 2.8 to 21.9°C with a mean of 13.18°C, and the mean annual rainfall ranges from 698.5 to 1083.5 mm with a mean annual of 920 mm [18]. Sheno is also the administrative center of Kembibit district in the north shoa zone, Oromia regional state, located 78 km away northeast of Addis Ababa, and it has an elevation of 2918 meters above sea level [18]. The altitude of the area ranges from 2620 to 3020 m.a.s.l and predominantly has Dega (highland cold agro-climatic zone) climate. The annual rainfall is 1013 mm, and the mean minimum and maximum temperatures of the area are 8°C and 20°C, respectively [18].

### 2.2. Sample Size and Sampling Methods

Four study districts, two kebeles from each district, for a total of eight kebeles, were selected based on the availability of crossbred dairy cows/heifers.

### 2.3. Dairy Farm Sample Size

The sample size of dairy farms, which were included in this investigation, was determined using the sample size willpower method, according to Arsham [19]. The sample size of dairy farms was determined as follows: this approach was operating with the assessment of 3% of standard error (SE), *n* = 0.25/SE2. 0.25/(0.03) (0.03)^2^, and a total of 277 dairy farms were identified; however, only 240 dairy farms were taken into account for this study, and the remaining 37 dairy farms were disqualified because they lacked the necessary information regarding their dairy cows or were unwilling to participate.

#### 2.3.1. Dairy Cows Sample Size

Using the Thrusfield [20] method, the sample size of dairy cows needed for this investigation was calculated, taking into account both the necessary absolute precision and the predicted percentage of repeat breeders. The sample size was calculated using a 95% confidence interval, a 5% desirable absolute precision, and a 50% predicted prevalence because there are no comparable reports in the study area.

(1) $$\begin{array}{c} N=\frac{1.96^{2}P\left(1-P\right)}{d^{2}}\text{,} \end{array}$$

where *P* = predicted occurrences 50%, *d* = required level of accuracy (5%), and *N* = Sample size.

(2) $$\begin{array}{c} N=\frac{1.96^{2}0.5\left(1-0.5\right)}{0.05^{2}}=384\text{.} \end{array}$$

One to five dairy cows per farm were nevertheless added to the study to boost its accuracy. As a consequence, 126 dairy cows were added to the sample of 384 dairy cows, making a total of 510 dairy cows. Thirty dairy farms out of a total of 240 dairy farms with at least 10 pregnant cows and heifers were chosen for the longitudinal study.

### 2.4. Study Design and Data Collection Methods

To determine the prevalence and incidence of RB, respectively, and its associated risk factors, two studies, one retrospective study, and the other longitudinal study using regular follow-up were conducted between December 2019 and May 2021.

#### 2.4.1. Retrospective Study

To investigate the prevalence of RB and associated risk factors in crossbred dairy cows, a retrospective study was used. To identify RB, predisposing reproductive diseases, and related risk factors, information from the record books and/or casebooks including the records of 510 distinct dairy cows held on 240 dairy farms was gathered. To fill in gaps in the record books of the dairy farms, interviews with the owners, managers, and custodians of the dairy farms were also undertaken. In crossbred dairy cows, data on the history of RB and predisposing reproductive issues (abortion, dystocia, RFM, metritis/endometritis, vaginal, and/or uterine prolapse) and their major risk factors (parity, milk yield, breeding practices, management conditions, feeding, health care, housing, sanitation) were meticulously collected from casebook records of the 510 individual dairy cows kept under 240 dairy farms. Herd size, management condition, production system, and techniques for detecting heat were some of the data that were carefully collected through visual observations and interviews with dairy farm owners. The dentition method and dairy farm birthdate records were also used to calculate the age of the dairy cows. To determine the nutritional status of all the dairy cows under investigation, body condition was measured. According to Richard [21], animals were categorized into body condition scores (BCS) of 0, 1, 2, 3, 4, and 5. To determine the impact of BCS on the occurrence of RB in crossbred dairy cattle, cows were divided into three categories based on their BCS: poor (≤2.5), medium (2.75 to 3.5), and good (≥3.75) [15].

Age and parity of cows were divided into three groups according to Nishi et al. [22] and Dulo et al. [23], namely age (≤4 years, 5 to 6 years, and ≥7 years) and parity (≥2nd, 3rd to 4th, and ≥5th parity), and their impacts on the prevalence of RB were assessed. Based on milk yield data collected during the study period, the milk yield of cows was also divided into three groups: 10 liters, 10 to 20, and 20 liters daily, and the impacts during RB were assessed. Production systems were categorized using Richard's approved criteria [21]. Extensive, semi-intensive, and intensive production systems were thus defined, and their impacts on the prevalence of RB in crossbred dairy cattle were evaluated. The following three classes were used by Nish et al. [22] to categorize the management conditions of dairy farms: Poor: cows were given conventional feed like grazing and limited straw feeding; they were kept in a typical floor without any facilities; no precautions were taken to keep the cows healthy or deworm them. Medium: Cows were given some concentrate and straw feeds, and they were kept on a farm with basic amenities such a concrete floor and manager that weren't scientifically designed and had a bad drainage system. They also periodically received irregular deworming and took steps to prevent sickness. Good: before and after calving, cows received balanced feed (concentrate, vitamin, and mineral mixture), including brewery by-product and straw; the cows were housed on a farm with a concrete floor, which was made relatively scientifically and has a good drainage system; the animals were dewormed and had regular disease prevention measures administered every two months. In a similar manner, their influences on the occurrences of RB were assessed.

#### 2.4.2. Longitudinal Study

In order to determine the incidence of RB and its risk factors in crossbred dairy cows/heifers, this study was designed by routinely monitoring dairy cows. To track the occurrence of RB and other predisposing reproductive problems, 186 dairy cattle from 13 dairy farms were periodically observed. The dairy cattle that were being evaluated were identified by their ID and parity, and they were routinely monitored throughout their pregnancies until parturition and for three months afterward by marking the time of parturition in their record books. Cows/heifers were closely observed throughout pregnancy and subsequent calving; their body fever was measured, and feed consumption was monitored. If the cow's fetal membrane was not removed or ejected within 24 hours of delivery, it was reported as RFM; if the cow was not conceived in three or more regularly spaced services recorded as RB, if the cow had a parturition issue, it was noted as dystocia, and if the fetus was born before the whole gestation time, it was noted as an abortion. Metritis/endometritis was diagnosed if the cow had vaginal discharge after parturition [24]. Last but not least, the relationships between the occurrence of RB and the underlying reproductive issues (abortion, dystocia, RFM, metritis or endometritis, and vaginal/uterine prolapse) were assessed.

#### 2.4.3. Body Condition Score

In order to evaluate the animals' nutritional status, the body condition of dairy cattle was examined two months prior to calving, at calving, and two months after calving. Then, in agreement with Richard [21], dairy cows were divided into BCS 0, 1, 2, 3, and 5. Cows were rated as poor (less than 2.5), medium (between 2.75 and 3.5), or good (above 3.75), and the impact on RB occurrences was assessed.

### 2.5. Statistical Analysis

Statistical Package for Social Science (SPSS) software 25 version was used to sort, compute, code, and analyze the data that had been collected. To calculate the prevalence and incidence of repeat breeding in percentages, descriptive statistical analysis was used. The chi-square (*X*^2^) test was used to investigate the relationship between RB and risk factors such as age, parity, BCS, milk yield, production system, management condition. Before being employed in the models, all independent continuous variables were converted into categorical variables. The potential for a univariate connection with repeat breeding was initially assessed for all risk factors. Then, in multivariate models of logistic regression, risk factors with univariate associations were evaluated (*P* < 0.05), and odds ratios (OR) with 95% confidence intervals (CI) were produced. The influence of each risk factor on the likelihood of repeat breeding in crossbred dairy cows/heifers was examined using odds ratios. In the final model, differences with *P* < 0.05 were regarded as significant. The comparison of the model's deviation to a *x*^2^ distribution was used to evaluate the model's goodness-of-fit.

## 3. Results

In a retrospective investigation, 510 crossbred dairy cattle were evaluated, and it was discovered that 38.4% of them had a prevalence of RB (Table 1). A longitudinal study of 186 crossbred dairy cattle discovered that 36.6% had an incidence of RB (Table 1).

### 3.1. Prevalence and Risk Factors of Repeat Breeding

This study recorded statistically significant differences and associations of the prevalence of RB (*P* < 0.05) with the age of dairy cattle. In comparison to cows under the age of four years, the odds ratio (OR) predicted for the prevalence of RB was 9.3 and 2.7 times greater (*P* < 0.05) in the age groups of cows over seven and five to six years, respectively. A statistically significant effect of dairy cow parity on the prevalence of RB was observed (*P* < 0.05. According to the results, the prevalence of RB was 3.3 and 4.8 times greater (*P* < 0.05) in dairy cows with parties of 3 to 4, and ≥5, respectively, than it was in cows with parties of 2 or lower.

Regarding dairy cows' bodily condition and milk production, there was a significant difference (*P* < 0.05) in the prevalence of RB (Table 2). In comparison to cows with strong BCS (≥3.75), odds ratio estimations showed that the prevalence of RB was 24.3 and 2.9 times greater (*P* < 0.05) in cows with poor (≤2.5) and medium (2.75 to 3.5) BCS, respectively. Additionally, compared to cows that generate fewer than 10 liters of milk per day, the prevalence of RB was 20.9 and 4.4 times higher (*P* < 0.05) in cows who produce 20 or more liters and 10 to 20 liters of milk per day, respectively. Breeding practices have an impact on RB prevalence as risk factors as well. The prevalence of RB was higher when using AI as a breeding method 25 (61.0%) than using natural and both breeding methods. In contrast to cows bred using both AI and natural breeding techniques, the odds ratio estimate for the prevalence of RB was 6.4 and 1.5 times greater (*P* < 0.05) in cows bred using AI and natural breeding techniques, respectively. The herd size and production system had a significant (*P* < 0.05) relationship with the prevalence of RB. The prevalence of recurrent breeding was found to differ significantly (*P* < 0.05) among production strategies and herd size categories.

It is highest in the extensive production system and large herd size (>20 herds) than intensive and semi-intensive production systems and small (<10 herds) and medium (10–20 herds), respectively (Tables 2 and 3). Estimates of the odds ratios showed that these risk factors were related to the prevalence of RB. As a result, RB prevalence was 9.4 and 2.9 times greater (*P* < 0.05) in dairy cattle managed under extensive and semi-intensive production systems, respectively, than in dairy cattle handled under the intensive production system. In comparison to dairy cattle found in small herd numbers, it was also 12.6 and 6.8 times greater (*P* < 0.05) in dairy cattle located in relatively big and medium herd sizes, respectively.

In Table 3, risk factors (management condition, heat detection practice, and time of AI) were revealed to have highly significant differences and associations with the prevalence of RB (*P* < 0.05).

According to the data, RB prevalence was 13.4 and 4.5 times greater (*P* < 0.05) in dairy cattle under bad and medium management settings than it was in those under good management conditions. Furthermore, the prevalence of RB was 13.4 and 42.5 times higher (*P* < 0.05) in dairy cow/heifers that had their heat signs properly detected (>20 minutes per detection time), was timely inseminated, respectively, when compared to dairy cattle that had their heat signs not properly detected (>20 minutes per detection time), and was not timely inseminated. Abortion, dystocia, RFM, metritis or endometritis, and vaginal/uterine prolapse were all substantially (*P* < 0.05) correlated with the occurrence of RB. In dairy cows predisposed to abortion, dystocia, RFM, metritis/endometritis, and vaginal/uterine prolapses, respectively, the prevalence of RB was 2.97, 2.01, 1.5, 2.0, and 3.8 times greater (*P* < 0.05) than in dairy cows who were negative for these reproductive issues.

### 3.2. Incidence and Risk Factors of Repeat Breeding

The incidence of RB and its risk factors in crossbred dairy cattle are shown in Table 4. According to the results of the present study, all risk factors, including age, parity, BCS, milk yield, breeding method, and management conditions, had extremely significant variations and are substantially related to the incidence of RB (*P* < 0.05). When compared to young-age dairy cows, older and adult-age dairy cows had greater occurrences of RB, which were 7.6 and 1.7 times higher (*P* < 0.05), respectively. The incidence of RB was 9.9 and 2.4 times (*P* < 0.05) greater in dairy cows with ≥5 and 3–4 parity, respectively, than in dairy cows with ≤2 parity. Considering the BCS, the incidence of RB was high in poor body conditioned cows (75%) compared to dairy cows with medium and good BCS. When compared to dairy cows with medium body condition ratings, RBS was discovered to be 9.7 times greater (*P* < 0.05) in poor-body-condition dairy cows.

This study found a statistically significant relationship between RB (*P* < 0.05) and rising milk yield, with the highest incidence found in cows producing 20 or more liters of milk per cow per day compared to those producing less than 10 and between 10 and 20 liters per cow per day. The incidence of RB in dairy cows bred by AI and natural service was 4.4 and 1.4 times higher (*P* < 0.05), respectively, than in dairy cows bred by the two methods of breeding. The impact of management conditions on the occurrence of RBS was found to be significantly related (*P* < 0.05). In dairy cattle with poor and medium management, respectively, the incidence of RB was 12.9 and 3.8 times greater (*P* < 0.05) than in dairy cattle with good management. The incidence of RB in crossbreed dairy cattle with risk factors (herd size, heat detection, time of insemination, and other predisposing reproductive disorders) is presented in Table 5.

Herd size had a substantial impact on the incidence of RB (*P* < 0.05). In comparison to dairy cattle with small herd numbers, the odds ratio predicted for the occurrence of RB was 2.0 and 1.1 times greater (*P* < 0.05) in dairy cattle with relatively large and medium herd sizes, respectively. This study demonstrated a statistically significant difference and a connection between RB and dairy cow insemination timing and heat detection practices (*P* < 0.05).

The incidence of RB was also 3.7 and 8.6 times higher (*P* < 0.05) in dairy cattle that had their heat signs properly detected (>20 minutes per detection time), was timely inseminated, compared to dairy cattle that had their heat signs not properly detected (<20 minutes per detection time), and was not timely inseminated. Abortion, dystocia, RFM, metritis/endometritis, and vaginal/uterine prolapse are predisposing reproductive issues that have statistically significant associations with the occurrence of RB (*P* < 0.05). In dairy cows prone to dystocia, RFM, metritis/endometritis, abortion, dystocia, and vaginal/uterine prolapses, respectively, the incidence of RB was substantially (*P* < 0.05) higher (54.8, 53.1, 56, 64.1, and 56.5%).

## 4. Discussion

In contrast to many studies that indicated a maximum prevalence of 26.8, 21.8, 15.9, and 28% [12, 14, 15, 25] in various regions of Ethiopia, respectively, the prevalence and incidence of RB in the present study were quite high, at 38.4% and 36.6%, respectively. On the other hand, the current investigation revealed a lower incidence of RB than occurrences in central Croatia/America, and tropical environments, which were correspondingly 42.4% and 62%, respectively [26, 27]. According to PerezMarin & Espana [28, 29], prevalence accounts for up to 37.8 and 41.8 percent of RB, respectively. The high occurrence of RB may be brought on by inadequate dietary control, ineffective insemination, wrong AI timing, and low semen quality [15]. Additionally, the variances may be a result of individual variation, study design, study location, study design in agroclimatic zones, and measurements employed to determine RB difference.

The age of the animals was found to have a statistically significant relationship with RB in the current investigation, with cows older than 7 years showing the highest prevalence and incidence of the disease. The results of a prior study [30] revealed that RB was more common in cows older than 7 years. Similar findings indicated that RB was substantially more prevalent in elderly dairy cows than in young and adult dairy cows [31]. The significant difference in reproductive problems with age is due to immunity as an animal's immunity declines with age, and it becomes more vulnerable and susceptible to reproductive disorders [32]. Furthermore, it is generally understood that age has a negative influence on fertility, with older cows having higher rates of RB [33]. This might be brought on by low hypothalamus and pituitary hormone levels or a decline in ovarian response capacity with advancing age [34]. In this investigation, the prevalence and incidence of RB substantially (*P* < 0.05) increased with parity in crossbred dairy cattle. This study agrees that multiparous cows have a higher prevalence of RB than primiparous cows [35]. Similar to past research that demonstrated multiparous cows have more postpartum reproductive problems than primiparous cows [36, 37] and that cow fertility declines as parity increases [38].

In the present investigation, dairy cows with low BCS showed significantly higher prevalence and incidence of RB (*P* < 0.05) than dairy cows with medium and good BCS. This is comparable to the findings of Esheti and Moges [15], who found an inverse relationship between decreasing body conditions and corresponding reproductive problems. Poorly maintained dairy cows are more likely to experience a negative energy balance, which impairs the release of reproductive hormones and increases the risk of fertilization failure or early embryonic death, both of which raise RB [39]. Milk yield had a statistically significant (*P* < 0.05) effect on the prevalence and incidence of RB. Due to changes in reproductive physiology [40] and an increase in the number of services per conception, higher milk supply capacity has been associated with lower fertility in dairy cows [41], leading to lower fertility rates [42].

The prevalence and incidence rate of RB were considerably greater (*P* < 0.05) in dairy cows/heifers bred using AI methods of service than in those bred naturally and using both methods. Similar to the current findings, the prevalence of infertility issues was higher in AI methods of service than in natural and both methods of service [31], which may be related to insemination technique, improper handling of infected semen, improper semen collection, or defective heat detection. The effect of herd size on the prevalence and incidence of RB significantly increased (*P* < 0.05) progressively from small to large herd size dairy farms. Similar to the previous finding that small and medium farms have lower rates of infertility than large farms. This could be accounted for by the fact that cows on small farms received more individualized care from the owner than those on large farms [44]. Additionally, farms with fewer dairy cows exhibited superior heat detection than farms with more dairy cows [45].

The prevalence rate of RB with the production system was strongly associated (*P* < 0.05) with extensive, semi-intensive, and intensive management systems, and it was much greater in dairy cattle with extensive management systems than those under semi-intensive and intensive systems. According to the results of the current study, the prevalence of RB was highest in crossbred dairy cows housed in extensive and semi-intensive management systems and lowest in intensive management systems [46]. This could be as a result of a dearth of records, inaccurate heat detection, poor nutrition, communal use of bulls for natural services, inadequate housing, and inadequate feeding management in the extensive production system [46]. When compared to dairy cows under medium and good management conditions, the prevalence and incidence rate of RB were considerably (*P* < 0.05) higher in cows under poor management condition. This result fairly agrees with previous findings, which recorded a higher prevalence of reproductive problems in poor management conditions such as poor cleanliness than in medium and good cleanness houses [31]. This could be as a result of the animal's dirty environment and unclean body making it more susceptible to microbial infection. Poor management including inadequate feeding management of the dairy cow may not fulfil the nutritional requirements of the lactating and pregnant cows leading to the animal going into negative energy balance, which resulted in decreased reproductive performance.

By preventing the production of luteinizing hormone, inadequate nutrition disrupts the restart of ovarian activity and the calving to conception interval and delays the occurrence of ovulation [47]. The farm's food management has a substantial impact on the occurrences of RB since; for instance, green grass supplementation aids in the follicular-genesis process, and carotene increases the conception rate in dairy cows [48]. Higher levels of oestradiol and progesterone may be caused by increased follicle size and corpus luteum functioning [49]. The findings of this study showed that there was a statistically significant relationship between the prevalence and incidence of RB in dairy cows with heat detection practices and artificial insemination timing (*P* < 0.05); the prevalence and incidence of RB were highest in dairy cows with incorrect heat detection and improper artificial insemination timing. These errors in heat detection and AI timing in dairy cows aggravate the problem of RB [50]. Furthermore, this study found that dairy cows with a history of reproductive issues (abortion, dystocia, RFM, metritis/endometritis, and vaginal/uterine prolepses) during the previous calving had considerably greater prevalence and incidence of RB (*P* < 0.05). Metritis (clinical and subclinical), abortion, dystocia, and retained placenta all exhibited a substantial relationship with RB [51, 52].

## 5. Conclusions and Recommendations

In comparison to other studies conducted in various regions of Ethiopia, the results of the current study showed significantly higher prevalence and incidence of RB in crossbred dairy cows/heifers, showing that RB occurs among crossbred dairy cows maintained in various management systems. However, the finding might indicate a fall in the proportion of cows replaced with first-calving heifers, as well as a reduction in milk production and calf sales revenue. The most sensitive cows to RB were discovered to be multiparous, old, with poor physical condition, high milk producers, and cows under large-scale farms. The occurrence of RB in the research area was highly influenced by poor management condition, AI methods of service, and predisposing reproductive issues (abortion, dystocia, RFM, metritis/endometritis, and vaginal/uterine prolapse). The following recommendations are made in light of the facts stated:

- Since this study indicated that cows and heifers with good body condition kept in appropriate housing conditions were less impacted by RB, dairy cattle producers should be recommended to enhance the management practices of dairy cattle, give adequate feed and health care, and properly manage housing
- The severity of RB issues may be reduced by adequate heat detection, timely insemination, and appropriate bull selection for breeding while taking into account the size of the cows
- To reduce RB and other predisposing reproductive problems, dairy producers should be trained in routine inspection, management, and handling of cows during postpartum periods.

## Figures and Tables

**Table 1:** Occurrences of repeat breeding in crossbred dairy cattle.

Method of study	No. animals examined	No. cow/heifers affected	(%)
Retrospective study	510	203	38.4
Longitudinal study	186	68	36.6
Total	696	271	38.9

**Table 2:** Repeat breeders with risk factors (age, parity, BCS, milk yield, breeding methods, and herd size) in crossbred dairy cattle.

Risk factors	Retrospective study					
	Group	No. of cows/heifers examined	Prevalence of RB no. (%)	*X* ^2^ (*P*-value)	Odds ratio (95% CI)	*P*-value
Age (years)	<4	201	32 (15.9)	136 (<0.01)	Reference	<0.01
	5-6	148	44 (29.7)		2.7 (1.2–6.4)	
	>7	161	120 (74.5)		9.2 (2.9–28.4)	

Parity (number)	<2	134	21 (15.7)	133.1 (<0.01)	Reference	<0.01
	3 to 4	221	58 (26.2)		3.3 (1.4–7.6)	
	>5	155	117 (59.7)		4.8 (1.6–15.1)	

BCS	Poor	159	115 (72.3)	43.15 (<0.01)	24.3 (10.2–57.9)	<0.01
	Medium	272	72 (26.4)		2.9 (1.3–6.7)	
	Good	81	9 (11.1)		Reference	

Milk yield (litters)	<10	148	33 (22.3)	37.84 (<0.01)	Reference	<0.01
	10 to 20	235	95 (40.4)		4.4 (2.5–7.8)	
	>20	126	74 (58.7)		20.9 (9.9–44.1)	

Breeding methods	AI	284	144 (50.7)	37.6 (<0.01)	6.4 (3.9–10.6)	<0.01
	Natural	146	45 (30.8)		1.5 (0.9–2.5)	
	Both	80	13 (16.2)		Reference	

Herd size (numbers)	<10	139	54 (38.8)	23.7 (<0.01)	Reference	<0.01
	10–20	74	40 (54.1)		6.8 (1.9–24.5)	
	>20	27	24 (88.9)		12.6 (3.6–43.8)	

**Table 3:** Prevalence of repeat breeding across with production system, feeding management, heat detection, time of AI, and predisposing reproductive problems) in crossbred dairy cattle.

Risk factors	Retrospective study					
	Group	Cows/heifers examined (no)	Prevalence no. (%)	*X* ^2^ (*P*-value)	Odds ratio (95% CI)	*P*-value
Production system	Extensive	73	51(69.9)	63.4 (<0.01)	9.4 (5.1–17.4)	<0.01
	Semi-intensive	239	106 (44.4)		2.9 (1.7–5.1)	
	Intensive	198	39 (20.2)		Reference	

Management condition	Poor	87	63 (72.4)	93.2 (<0.01)	13.4 (7.4–24.4)	<0.05
	Medium	215	100 (46.5)		4.5 (2.8–7.0)	
	Good	208	33 (15.9)		Reference	

Heat detection	≤20 min/detection time	90	77 (85.6)	102.6 (<0.01)	13.4 (7.3–24.6)	<0.01
	≥20 min/detection time	420	119 (28.3)		Reference	

Time of AI	Correct	396	104 (26.3)	162.9 (<0.01)	Reference	<0.01
	Incorrect	114	92 (80.7)		42.5 (15.2–119.9)	

*Predisposing reproductive problems*						
Abortion	Yes	70	43 (61.4)	17.8 (<0.01)	2.97 (1.8–5.0)	<0.01
	No	440	154 (35.0)		Reference	
Dystocia	Yes	62	31 (50.0)	3.8 (<0.05)	2.01 (1.2–3.6)	<0.01
	No	448	166 (37.1)		References	
RFM	Yes	149	72 (48.3)	8.3 (<0.01)	1.5 (1–2.2)	<0.05
	No	361	125 (34.6)		References	
Metritis/endometritis	Yes	96	53 (55.2)	13.7 (<0.01)	2.0 (1.3–3.2)	<0.01
	No	414	144 (34.8)		References	
Vaginal/uterine prolapses	Yes	33	25 (75.8)	20.5 (<0.01)	3.8 (1.6–9.1)	<0.01
	No	477	172 (36.1)		References	

**Table 4:** Incidence of repeat breeding with risk factors (age, parity, BCS, milk yield, breeding methods, hygiene, and feeding management in crossbred cow/heifers.

Risk factors	Longitudinal study					
	Group	Cow/heifers studied (no)	Incidence no. (%)	*X* ^2^ (*P*-value)	Odds ratio (95% CI)	*P*-value
Age (years)	<4	75	15 (20.0)	29.6 (<0.01)	References	<0.01
	5-6	56	17 (30.4)		1.7 (0.78–3.89)	
	>7	118	68 (57.6)		7.6 (3.43–16.75)	

Parity	<2 parity	100	21 (21.1)	32.8 (<0.01)	References	<0.05
	3–4 parity	46	18 (39.1)		2.4 (1.13–5.19)	
	>5	40	29 (72.5)		9.9 (4.26–23.08)	

BCS	Poor	44	33 (75.0)	37.0 (<0.01)	9.7 (4.39–22.18)	<0.01
	Medium	99	23 (23.2)		Reference	
	Good	43	12 (27.9)		1.1 (0.43–2.65)	

Milk yield	<10 litters	31	6 (19.4)	18.6 (<0.01)	Reference	<0.01
	10–20 litters	90	25 (27.8)		1.6 (0.59–4.37)	
	>20 litters	65	37 (56.9)		5.5 (1.99–16.23)	

Breeding methods	AI	93	46 (49.5)	13.7 (<0.01)	4.1 (1.43–11.83)	<0.05
	Natural	67	17 (25.4)		1.4 (0.47–4.38)	
	Both	26	5 (19.5)		Reference	

Management condition	Poor	56	33 (58.9)	20.2 (<0.01)	12.9 (2.73–61.14)	<0.01
	Medium	110	33 (30.0)		3.8 (1.71–6.55)	
	Good	21	3 (10.0)		Reference	

**Table 5:** Incidence of repeat breeding with risk factors (herd size, heat detection, time of insemination, and predisposing reproductive disorders) in crossbred dairy cattle.

Risk factors	Retrospective study					
	Group	Cows/heifers studied (no)	Incidence no. (%)	*X* ^2^ (*P*-value)	Odds ratio (95% CI)	*P*-value
Herd size (number)	10–15	30	8 (26.7)	4.4 (<0.01)	Reference	<0.05
	15–25	43	12 (27.9)		1.1 (0.4–3.0)	
	>25	113	48 (42.5)		2 (0.8–4.9)	

Heat detection	Daily ≤20 min/detection time	68	38 (55.9)	17.3 (<0.01)	3.7 (1.9–6.9)	<0.01
	Daily ≥20 min/detection time	118	30 (25.4)		Reference	

Time of AI	Correct	131	29 (22.1)	39.7 (<0.01)	Reference	<0.01
	Incorrect	55	39 (70.9)		8.6 (4.2–17.5)	

*Predisposing reproductive disorders*						
Abortion	Yes	52	23 (44.2)	7.7 (<0.05)	2.7 (1.3–5.4)	<0.05
	No	144	45 (31.3)		Reference	
Dystocia	Yes	32	17 (53.1)	4.6 (<0.05)	2.3 (1.1–4.9)	<0.05
	No	154	51 (33.1)		Reference	
RFM	Yes	50	28 (56.0)	11.1 (<0.01)	2.2 (1.1–4.6)	<0.05
	No	136	40 (29.4)		Reference	
Metritis/endometritis	Yes	31	20 (64.5)	12.5 (<0.01)	2.9 (1.2–6.9)	<0.05
	No	155	48 (31.0)		Reference	
Vaginal/uterine prolapse	Yes	23	13 (56.5)	4.5 (<0.03)	2.6 (1.1–6.2)	<0.05
	No	163	55 (33.7)		Reference	

## Data Availability

The evidence cited to support the study's findings is contained in the article and is also available upon request from the authors.
